# Vomiting and Its Treatment

**Published:** 1888-12

**Authors:** R. C. Word

**Affiliations:** Professor of Physiology in the Southern Medical College, Atlanta, Ga.


### Southern Medical Record.

A MONTHLY JOURNAL OF PRACTICAL MEDICINE.

☞ All Communications must be addressed to R. C. WORD, M. D., Managing Editor

"*Quicquid Præcipies Esto Brevis.*"

VOL. XVIII. ATLANTA, GA., DECEMBER, 1888. No. 12.

### ORIGINAL AND SELECTED ARTICLES.

### VOMITING AND ITS TREATMENT.

ABSTRACT OF A LECTURE BY R. C. WORD, M. D.
Professor of Physiology in the Southern Medical College, Atlanta, Ga.

The word vomit is from the Latin *vomo*, which means to eject the contents of the stomach. Nausea is an almost invariable accompaniment of the act of vomiting. By nausea is meant sick stomach. It is derived from the Greek word *naus*, a ship, having reference to sea sickness, so common to those who for the first time go to sea.

Vomiting is a compound act, by which I mean that a number of agencies contribute to it. The diaphragm and abdominal muscles are the principal forces brought into action. It is always preceded by a deep inspiration, the glottis is closed, the cardiac sphincter is relaxed, the pylorus is closed, the peristalsis is reversed, the stomach contracts, co operating with the action of the abdominal muscles, and so the contents are forcibly ejected, accompanied, and usually preceded, by a profuse watery secretion from the salivary and buccal glands.

Vomiting is a physiological act, a process by which the economy seeks to empty the stomach of offending matters.

There is a nerve centre which presides over the act of vomiting. This centre has been located in the medulla oblongata, and the sensory or afferent nerves which convey the impression to the nerve centre are the pneumogastric the fifth, and the glosso-pharyngeal, the influence being reflected through efferrent nerves upon the stomach and the muscles concerned in the act. The sensory nerves from the uterus, from the kidneys, the testicles and from other organs may impress the nerve centre and produce vomiting. The centre may also be impressed by influences from the cerebrum and cerebellum, as where disgusting sights, or thoughts of nauseous medicines excite it.

The efferent, or outgoing impressions, are conveyed to the stomach and adjacent parts by the phrenic and the spinal nerves.

Vomiting is comparatively easy in children, especially in infants, because of the undeveloped condition of the cul-de-sac at the cardiac orifice, and also by reason of the smallness of the fundus or larger curvature of the organ.

In any case which is due to a simple local irritation, as from an emetic or some offensive drug or foreign matter taken into the stomach, the vomiting usually ceases when the irritating cause is ejected. Frequently, however, especially in malarious regions, the presence of bile leads to a persistent and distressing vomiting. Fresh bile from the liver, when from torpor, congestion, constipation or other trouble in the duodenum, it finds its way into the stomach, is an exceedingly nauseating and active emetic.

*Emetics* may be classed as of two kinds, *local* and *general.* Ipicac probably acts as a local irritant to the mucous membrane of the stomach. There are certain agents which act as chemical irritants, as do the sulphate of zinc, sulph. copper, alum, etc. Mustard acts probably by its local irritating effect upon the gastric mucous membrane. As an example of the power of mere local influence without chemical agency, I mention the fact that the application of a feather, or other foreign substance to the fauces invariably excites the nerve centre of vomiting.

Vomiting may result from many influences, and many diseased conditions, even in organs remote from the stomach. We often see it in diseases of the kidneys, testicles, the bladder, the womb; in constipation, obstruction of the bowels, stone in the gall-duct and ureter, in cholera, cholera infantum, from violent blows or concussions of the head, etc.

General emetics are those which affect the nerve centre of vomiting through absorption into the circulation. An instance of this property is found in apomorphia, one-fourth of a grain of which injected hypodermatically will cause prompt and efficient emesis. It is an important agent to produce vomiting in cases of opium poisoning, or in other conditions in which the patient can not swallow.

But my purpose to-day is not so much to describe the causes, or the varied and intricate reflex influences which may contribute to the act of vomiting, as to give you some practical hints touching the treatment of vomiting in those cases which, from any cause, the nerve centre may become morbidly excited so that the vomiting continues to a degree which is excessive and sometimes uncontrollable.

In all cases where vomiting becomes troublesome, it is well first to ascertain, if possible, the cause of the trouble. If there is any reason to believe that all offending matters have not been ejected from the stomach, it will be well to give the patient tepid water to encourage the vomiting for a time until satisfied that the stomach is cleared of its contents. Then as a rule let a sinapism be applied to the epigastric region, and let the patient have small lumps of ice frequently administed, to dissolve in his mouth, the cool ice water being swallowed. After the sinapism is removed, apply a cloth wet with mint water, or make an application of garden mint bruised to the epigastrium. The ice treatment is particularly applicab'e in gastric inflammation, in which case a blister may follow the sinapism instead of the mint as above mentioned.

Few remedies internally will avail to allay the vomiting in obstinate cases. Yet I will mention those which have been found most available.

℞ Com. tinc. iodine ............................ gtt. xvi,

Water ............................................ ℥ ij.

M. S.—One teaspoonful every half hour.

The following, known as the Randolph mixture, is strongly recommended:

℞ Creosoti ........................................ gtt. 20,
Acetic acid ...................................... gtt. 40,
Sulph. morphine ............................... grs. 2,
Water ............................................ ℥ 2.

M. S.—A teaspoonful every half hour for two or three doses.

Or the following:

℞ Carbolic acid .................................. gtt. 1,
Chloroform ..................................... gtt. 3,
Alcohol ........................................... gtt. 20,
Water ............................................ ℥ ½.

M. S.—In one dose, to be repeated in half hour if need be. This is especially useful in cholera morbus.

Another well adapted to cholera infantum is:

℞ Carbolic acid............................gtt. 24,

Alcohol.................................gtt. 24,

Mint water................................℥ 1½,

Mucil. accaciæ, syrup popies.............aa gtt. 15.

M. S.—Give a teaspoonful every two hours. Apply at the same time upon the stomach of the child a plaster or poultice containing cloves, spice, ess. peppermint and ginger.

It is well in such cases also to give ¼th or ⅛th gr. calomel upon the tongue for a few doses, and wash down with a little ice water.

Of late, in the northern cities, in cases of cholera infantum, repeated small doses of ice water, with no other medicine, has been used with good results in many instances.

In the *cholera morbus* of adults, no remedy has given such good result as hypodermic morphia. Often I have relieved the most violent cases with a simple injection of ¼th grain of morphine. And I have also found the same remedy very efficient in the vomiting of sick headache, and other forms of violent and persistent vomiting. Indeed, I regard it as the sheet anchor in almost every phase of vomiting. In the milder cases I usually first try some one of the foregoing agents, and failing with them, I resort to the hypodermic morphia. In most cases I apply a sinapism to epigastric region before administering the morphia.

In the vomiting from pregnancy, I have been accustomed to use an opiate by enema in conjunction with bromide of potash as follows:

℞ McMunn's elix. opii.........................gtt. 30,
Brom. pot.....................................grs. 30,

Inject into the rectum in 2 ounces of warm water.

This remedy is usually followed by releif for a day or night, and may be repeated from time to time, but must be suspended every two or three days to open the bowels. In some cases the bromide used alone without the opium has given relief; and may be continued for a long while without injury.

The *oxalate of cerium* 1 to 5 grains three times a day, is a favorite remedy with some practitioners, and occasionally gives relief. A recent writer urges the use of larger doses of the oxalate of cerium as being safe and more efficient, 8 to 10 grains every one to three hours.

When such cases are extremely bad and persistent they may be often relieved by cauterizing the os uteri, or as has been asserted by some practitioners, by slightly dilating the os uteri with the finger, as the vomiting in such cases is due to a reflex influence, having its origin in the womb. The dilatation should be made by the index finger introduced within the neck, but not beyond the internal sphincter.

Dujarden-Beaumetz claims to have relieved the most obstinate cases of vomiting in pregnancy with the following prescription:

℞ Cocaine hydrochloratis.......................grs. vij,
Aquæ distil...............................℥ x.

M. S.—Dose one to two tablespoonsful every hour. The patient should lie down to prevent vertigo.

Recently irrigations of sulphuric ether to the epigastrium have been recommended as a superior remedy in extreme cases of vomiting, whether from pregnancy or other causes.

A medical friend informs me of remarkable success in the treatment of an extremely obstinate case of vomiting of pregnancy by injecting into the rectum a teaspoonful of bromidia diluted with a tablespoonful of water. This preparation contains chloral, bromide of potass., extract cannabis indica and hyoscyamus.

In many cases of extreme vomiting, whether from pregnancy or other causes, relief has sometimes followed the use of iced champagne, and effervescing drinks, and the froth from soda powders, etc.

Recently the nitrate of potas. ¼th grain oft repeated, has been suggested as a superior anti-emetic, also the acetate of lead ½ to 1 gr. every half hour for a few doses.

The Homœopathists place great stress upon small doses of certain nauseants, as 1 drop of the tinc. ipecac every fifteen or twenty minutes, or the 50th of a grain tartar emetic used in the same way.

I have found better results from tincture nux vomica in one drop doses oft repeated in that class of cases resulting from the after effects of opium or other drugs, from excesses in diet, drink or like causes. Here there exists a relaxed and enfeebled condition of the stomach, a want of tone, which may be sometimes benefitted by the invigorating effects of nux upon the nerve centre of vomiting, or it may act upon the peripheral terminations of the sensory nerves in the stomach, upon which also a good impression may sometimes result from the alterative influence of minute doses of ipecac and like agents.

I have seen, in some cases, a sudden cessation of a long continued vomiting spell from drinking a full cup of very hot coffee or full glass of cold buttermilk.

I have thus glanced briefly at the more important practical agents which have been found useful in excessive and obstinate vomiting, a condition which, as young practitioners of medicine, you will find among the most troublesome and embarrassing in your professional life.

